# Molecular characterization of phenylketonuria patients from the North Region of Brazil: State of Pará

**DOI:** 10.1002/mgg3.2224

**Published:** 2023-07-08

**Authors:** Pedro E. Bonfim‐Freitas, Roseani S. Andrade, Ândrea K. Ribeiro‐dos‐Santos, Luiz C. Santana‐da Silva

**Affiliations:** ^1^ Laboratory of Inborn Errors of Metabolism Institute of Biological Sciences, Federal University of Pará Belém Brazil; ^2^ Faculty of Nutrition Institute of Health Sciences, Federal University of Pará Belém Brazil; ^3^ Laboratory of Human and Medical Genetics Institute of Biological Sciences, Federal University of Pará Belém Brazil; ^4^ Present address: Hepatology Department Evandro Chagas Institute Belém PA Brazil

**Keywords:** genotype‐phenotype, north of Brazil, pathogenic variants, phenylketonuria, PKU

## Abstract

**Background:**

Phenylketonuria (PKU) is an autosomal recessive disease resulting from a deficiency of the enzyme phenylalanine hydroxylase (PAH). Hyperphenylalaninemias (HPA) due to PAH deficiency are accompanied by a wide variety of clinical, biochemical, and molecular features. To identify and characterize pathogenic variants in the PAH gene and establish a correlation between genotype and biochemical phenotype in patients with PKU from state of Pará in the North Region of Brazil.

**Methods:**

All 13 exons of the PAH gene from 32 patients (21 PKU and 11 non‐PKU HPA) were amplified by PCR and submitted to DNA sequencing (Sanger). Biochemical data were obtained from the patients' medical records.

**Results:**

Molecular analysis identified 17 pathogenic variants and 3 nonpathogenic variants. The most frequent pathogenic variants were IVS10‐11G>A (7.9%), p. Arg261Gln (7.9%), p. Val388Met (6.3%) and p. Ile65Thr (4.7%). Was observed correlations and inconsistencies between genotype and biochemical phenotype.

**Conclusion:**

In PKU patients from state of Pará, North Region of Brazil, a heterogeneous mutation spectrum was revealed, in which the most frequent mutations are variants commonly observed in other Brazilian studies and in the region of the Iberian Peninsula.

## Abstract 2

Molecular analysis performed identified 17 pathogenic mutations. The most frequent pathogenic variants were IVS10‐11G>A (7.9%), p. Arg261Gln (7.9%), p. Val388Met (6.3%) and p. Ile65Thr (4.7%). Was observed correlations and inconsistencies between genotype and phenotype.

## 1 INTRODUCTION

The main cause of hyperphenylalaninemia (HPA) is the deficiency of the hepatic enzyme phenylalanine hydroxylase (PAH, EC 1.14.16.1), which causes a blockage in the phenylalanine (Phe) degradation pathway, resulting in an accumulation of this amino acid in tissues and blood. HPA caused by PAH deficiency is commonly referred to as phenylketonuria (PKU; OMIM 261600) and has an autosomal‐recessive inheritance pattern (Blau et al., 2010; Scriver & Kaufman, 2001). This genetic disease presents with a wide variety of clinical, biochemical and molecular features, ranging from severe forms (classic PKU), characterized by the presence of mental deficiency, to moderate and mild forms, including a clinical condition known as non‐PKU hyperphenylalaninemia (non‐PKU HPA), which is generally benign and permits normal cognitive development (Blau et al., 2011, 2014; Willians et al., 2008).

The human gene *PAH* (OMIM: 612349) is located on chromosome 12, region 12q23 (Lidksy et al., 1984), is composed of 13 exons and covers approximately 100 kb of genomic DNA. The accession number for the *PAH* is RefSeq: ENSG00000171759; GeneBank: NM_000277.1. At present, more than 1000 variants have been identified in the *PAH* gene and registered in the *PAH*vdb – Phenylalanine Hydroxylase Gene Locus‐Specific Database (http://www.biopku.org/home/pah.asp). This high molecular heterogeneity may be associated with the wide clinical and biochemical spectrum observed in patients with PKU (Neto et al., 2019; Okano et al., 1991; Scriver & Kaufman, 2001). The distribution and relative frequency of mutations that impact PAH as well as the association between those mutations and certain haplotypes have already been described for several populations, highlighting notable interpopulation differences (Scriver, 2007; Zschocke, 2003).

The prevalence of the disease varies in diverse populations and regions worldwide, with an estimated PKU global prevalence of 1:23.930 in newborns (Hillert et al., 2020). In Brazil, the incidence of PKU has been reported to be approximately 1 in 25,000 live births, although there are important variations between Brazilian states, from 1:9000 to 1:33,000 (Neto, Filho, et al., 2018). There are several ways to classify PKU. The criteria for a diagnosis of PKU are generally based on the plasma Phe concentration at diagnosis (patient still untreated), Phe tolerance and the degree of PAH deficiency (Blau et al., 2011).

Mutational analysis in PKU becomes important, among other reasons, due to the possibility of an association between the genotype and the phenotype. In this way, by knowing the genotype of the patients soon after the diagnosis, it would be possible to make predictions regarding their prognosis and better plan the treatment (Blau et al., 2014; Gundorova et al., 2019; Zschocke, 2010).

Currently, the molecular data of PKU patients from the North Region of Brazil are still scarce since most of the molecular studies were carried out in the South, Southeast and Northeast Regions of Brazil. Therefore, there is little knowledge about the genotype–phenotype correlation in these patients. The present study aimed to identify pathogenic variants in the *PAH* gene and establish a correlation between genotype and biochemical (metabolic) phenotype in patients with PKU from the state of Pará in the North Region of Brazil.

## 2 MATERIALS AND METHODS

### 2.1 Patients

This study was based on the analysis of a sample composed of 32 patients diagnosed with HPA who were undergoing treatment or follow‐up at the Reference Service for Neonatal Screening in the state of Pará, located in the city of Belém in the North Region of Brazil. All families of patients who agreed to participate in this study received a written informed consent form in addition to information about the objectives of this research.

The fundamental criterion to include patients in the sample was the confirmed biochemical diagnosis of HPA (a Phe level >4 mg/dL) through the measurement of Phe in plasma performed within the national neonatal screening program. The total levels of Phe in plasma were analyzed using blood samples in dried blood spots. The families of newborns who exhibited high levels of Phe were contacted, and these newborns were reevaluated (clinical and biochemical evaluation) to confirm the diagnosis. For newborns with a confirmed diagnosis of HPA, treatment was based on dietary restriction of Phe. The patients diagnosed with non‐PKU HPA were regularly followed up, however, without the necessity of dietary therapy.

Patients with suspected abnormalities in the metabolism of the enzymatic cofactor BH_4_ were excluded from the study. In the present study, the levels of Phe before the treatment (at diagnosis) were used to characterize the PKU form of each patient. Patients who showed Phe levels above 20 mg/dL (>1200 μmol/L) before treatment were characterized as having classic PKU (*n* = 9). Patients with variations in pretreatment Phe levels between 10 and 20 mg/dL or 600 and 1200 μmol/L were characterized as having moderate/mild forms of the disease (*n* = 12). Patients with a Phe level <10 mg/dL (<600 μmol/L) were classified as having non‐PKU HPA (*n* = 11).

### 2.2 Molecular analysis

For the identification and molecular characterization of mutations, all 13 exons (including intron‐exon junctions) of the *PAH* gene were amplified by PCR using previously described primers and thermocycling conditions (Silva et al., 2003). For PCR, 10 pmol each primer was added to 100 ng template DNA with 1–5 U *Taq* DNA polymerase (Thermo‐Fisher, Massachusetts, USA), in a total volume of 25 μL containing dNTPs (2 mM), MgCl_2_ (50 mM), 10x PCR buffer and nuclease‐free ultra‐pure water. Genomic DNA was extracted from peripheral whole blood collected with EDTA using the phenol‐chloroform extraction method. All amplicons were analyzed by horizontal electrophoresis in 1.5%, 2.0% or 3.0% (p/v) agarose gels according to the size of the amplified region (170–324 base pairs). After amplification and confirmation, the PCR products were subjected to direct DNA sequencing using the Sanger method using the ABI PRISM BigDye Terminator v3.1 Cycle Sequencing kit (Applied Biosystems, Foster City, CA, USA). The sequencing reactions were performed in an ABI‐PRISM 377 model automatic sequencer (Applied Biosystems).

### 2.3 Biochemical data evaluation

Biochemical aspects (Phe level at the time of diagnosis and Phe measurements during treatment) data were obtained through a retrospective review of the medical records of patients undergoing treatment or follow‐up by the multidisciplinary team of Health of the Neonatal Screening Reference Service in the state of Pará. Patients are regularly monitored for laboratory evaluation (the quantitation of plasma Phe by fluorimetry), neuropsychomotor development and the adequacy of the dietary treatment prescription. Biochemical evolution (Phe levels during treatment) was assessed up to 5 years of age.

### 2.4 PAH residual activity (PRA)

Quantifications of PAH activity obtained from in vitro expression studies of normal and mutant *PAH* cDNA were used to predict the level of PAH activity in patients with known genotypes. The values of PAH residual activity (PRA) were obtained from a query in *PAH*vdb (http://www.biopku.org/home/pah.asp), in which residual enzymatic activities related to some variants are registered, and through a search of the literature. The PRA value was calculated considering the mean of the PAH activity levels associated with each mutant enzyme and was expressed as the percentage of the normal level of PAH activity.

### 2.5 Statistical analysis

The BioEstat program version 5.0 was used for statistical analysis. A *p*‐value ≤0.05 was considered statistically significant in all analyses. The binomial test was used to assess clinical data and compare the frequencies of mutations in the present study with those of other studies; the Mann–Whitney test was used for the comparisons of Phe levels and the comparisons of PRA values; Pearson's linear correlation was used to determine the correlation between PRA values and Phe levels. The frequencies of variants were determined by dividing the number of alleles carrying them by the total number of alleles searched. To calculate the percentage values of frequencies, this coefficient was multiplied by 100.

## 3 RESULTS

Regarding characteristics, of the 32 patients selected for this study, 53.12% were female and 46.88% were male. Patients between 1 and 12 years of age constituted 87.5% of the studied sample, and 12.5% were older than 12 years. Regarding the origin (the city of origin), 28.13% came from the capital Belém and 71.87% of the patients came from other cities in the state of Pará. The diagnosed patients came from 20 different cities in the state of Pará: Belém (11), Monte Alegre (1), São Miguel do Guamá (3), Altamira (1), Tucuruí (2), Marabá (1), Santarém (1), Conceição do Araguaia (1), Castanhal (1), Capanema (1), Eldorado dos Carajás (1), Novo Progresso (1), Oriximiná (1), Curralinho (1), Irituia (1), Brejo Grande do Araguaia (1), Breves (1), Mojú (1), Uruará (1) and Tomé‐Açu (1). An early diagnosis was made in 93.75% of the patients in the sample. In patients diagnosed early, the average biochemical diagnostic period was 20 days. The presence of consanguinity between parents was observed in 9.37% of the sample.

Molecular analysis performed on the DNA of 32 patients with HPA in the state of Pará identified 17 pathogenic variants and 3 nonpathogenic variants These variants were distributed in exons 3, 5, 6, 7, 10, 11 and 12 and in introns 2 and 10 of the *PAH* gene. All identified variants have already been described in previous studies. The nonpathogenic variants IVS2nt19T>C (intron 2), p.Q232Q (exon 6) and p.V245V (exon 7) presented allelic frequencies of 9.3%, 4.6% and 7.8%, respectively. The most frequent pathogenic variants in this study were IVS10‐11G>A (7.9%), p. Arg261Gln (7.9%), p. Val388Met (6.3%) and p. Ile65Thr (4.7%).

Table 1 describes some characteristics of all pathogenic variants identified in the present study. Variants associated with the in vitro enzymatic phenotype (PRA) of classic PKU (p. Arg158Gln, p. Glu280Lys, p. Thr278Ile, IVS10‐11G>A and p. Arg408Trp) or moderate PKU (p. Ile65Thr, p. Arg261Gln, p. Leu348Val and p. Val388Met) represented 52.9% of the total pathogenic variants. Five variants (29.4%) were associated with a mild PKU or non‐PKU HPA phenotype (p. Tyr414Cys, p. Asp415Asn, p. Ala403Val, p. Pro69Ser and p. Leu249Phe) and 17.6% (p. Asp84Tyr, p. Leu242Phe and IVS10+1G>A) were not associated with any enzyme phenotype due to the lack of in vitro expression studies for these variants.

**TABLE 1:** Pathogenic variants were identified in patients with HPA (PKU and non‐PKU HPA) from state of Pará, north of Brazil.

Nucleotide change	Protein variant	*PAH* gene region	Protein domain	Type	Relative frequency (%)	In vitro PAH activity (%)^a^
c.194 T>C	p. Ile65Thr	Exon 3	Regulatory	Missense	4.7	33
c.250G>T	p. Asp84Tyr	Exon 3	Regulatory	Missense	1.5	–
c.205C>T	p. Pro69Ser	Exon 3	Regulatory	Missense	1.5	69
c.473G>A	p. Arg158Gln	Exon 5	Catalytic	Missense	1.5	10
c.745C>T	p. Leu249Phe	Exon 7	Catalytic	Missense	3.1	51
c.724C>T	p. Leu242Phe	Exon 7	Catalytic	Missense	1.5	–
c.838G>A	p. Glu280Lys	Exon 7	Catalytic	Missense	1.5	11
c.833C>T	p. Thr278Ile	Exon 7	Catalytic	Missense	1.5	1
c.782G>A	p. Arg261Gln	Exon 7	Catalytic	Missense	7.9	44
c.1066‐11G>A	IVS10‐11G>A	Intron 10	–	Splicing	7.9	5
c.1065+1G>A	IVS10+1G>A	Intron 10	–	Splicing	1.5	–
c.1042C>G	p. Leu348Val	Exon 10	Catalytic	Missense	1.5	25
c.1162G>A	p. Val388Met	Exon 11	Catalytic	Missense	6.3	28
c.1241A>G	p. Tyr414Cys	Exon 12	Oligomerization	Missense	1.5	57
c.1222C>T	p. Arg408Trp	Exon 12	Catalytic	Missense	1.5	2
c.1243G>A	p. Asp415Asn	Exon 12	Oligomerization	Missense	1.5	72
c.1208C>T	p. Ala403Val	Exon 12	Catalytic	Missense	1.5	66
	Total				49.2%	

^a^ Enzyme activity: Residual PAH activity of the transiently expressed recombinant mutant proteins, compared with the wild‐type PAH. Average of percentage activities reported for different cells. (http://www.biopku.org/home/pah.asp).

Based on the identification of variants, it was possible to compare the PRA (%) between PKU patients and non‐PKU HPA patients. The comparison of PRA between PKU (mean = 20.2%) and non‐PKU HPA (mean = 37.5%) patients showed a statistically significant difference (*p* = 0.0487). According to the PRA values, a greater number of variants associated with the severe in vitro biochemical phenotype was observed in PKU patients, while in non‐PKU HPA patients, the presence of mild and moderate variants was identified. Regarding the distribution of pathogenic variants identified in this study in the PKU and non‐PKU HPA phenotypic groups, 12 variants were observed only in the PKU group (p. Arg408Trp, p. Tyr414Cys, p. Leu348Val, IVS10+1G>A, IVS10‐11G>A, p. Thr278Ile, p. Glu280Lys, p. Leu242Phe, p. Leu249Phe, p. Arg158Gln, p.Pro69Ser and p. Asp84Tyr), two variants only in the non‐PKU HPA group (p. Ala403Val and p. Asp415Asn) and three variants observed in the PKU and non‐PKU HPA groups (p.Val388Met, p. Arg261Gln and p. Ile65Thr).

Regarding the genotype, in nine patients (28.1%) of the sample, it was possible to identify the two mutant alleles. In 13 patients (40.6%), only one mutant allele was identified and in 10 patients (31.2%), no variants were identified. The frequency of heterozygous composite individuals was 77.7% (7/9), while the frequency of homoallelic genotypes was 22.3% (2/9). In patients with known genotypes (2 identified mutant alleles), it was possible to predict the level of PAH activity and establish a correlation with Phe levels.

This analysis was performed in six patients with a defined genotype, c. [IVS10‐11G>A]; p. [Tyr414Cys], c. [IVS10‐11G>A]; [IVS10‐11G>A], p. [Ile65Thr]; [Val388Met], c. [IVS10‐11G>A]; [IVS10‐11G>A], p. [Ala403Val]; [Val388Met] and p. [Arg261Gln]; [Asp415Asn], since in these patients it was possible to obtain the PRA (%) values for these variants. The evaluation of the correlation between the PRA (%) values and the pretreatment (*p* = 0.0165) and in‐treatment (*p* = 0.0115) Phe levels showed that there was a statistically significant correspondence. This correspondence was inversely proportional, as the correlation coefficients had negative values: pretreatment (*r* = −0.8932) and treatment (*r* = −0.9112). Table 2 shows the genotypes and the correlation between plasma Phe levels and PRA values in the patients analyzed.

**TABLE 2:** Correlation between biochemical parameter (pre‐treatment and in‐treatment Phe level) and PRA values in patients with HPA due to PAH deficiency from the state of Pará, Brazil.

Patients	Genotypes [allele 1]; [allele 2]	PRA (%)	Phe diagnosis (mg/dL)	Phe^a^ treatment (mg/dL)	Metabolic phenotype
1	c. [IVS10‐11G>A]; p. [Tyr414Cys]	31	24.4	10.7 ± 6.3	Classic PKU
2	p. [Leu242Phe]; [p. Arg158Gln]	?/10	24	11.3 ± 8.1	Classic PKU
3	c. [IVS10‐11G>A; [IVS10‐11G>A]	5	15	–	Moderate/mild PKU
4	c. [IVS10+1G>A]; p. [Pro69Ser]	?/69	9.5	11.1 ± 3.7	Moderate/mild PKU
5	c. [IVS10‐11G>A; [IVS10‐11G>A]	5	39	20.4 ± 10.3	Classic PKU
6	p. [Asp84Tyr]; [Thr278Ile]	?/1	6.2	8.0 ± 7.9	Moderate/mild PKU
7	p. [Ile65Thr]; [Val388Met]	34.5	18.2	10.1 ± 4.3	Moderate/mild PKU
8	p. [Leu249Phe]; –	51/?	7.4	5.9 ± 3.2	Moderate/mild PKU
9	p. [Leu249Phe]; –	51/?	7.8	10.2 ± 4.2	Moderate/mild PKU
10	p. [Arg261Gln]; –	44/?	10.8	11.4 ± 4.9	Moderate/mild PKU
11	p. [Arg408Trp]; –	2/?	14	–	Moderate/mild PKU
12	p. [Val388Met]; –	28/?	12.9	12.9 ± 3.6	Moderate/mild PKU
13	p. [Arg261Gln]; –	44/?	28.1	16.8 ± 7.4	Classic PKU
14	p. [p. Glu280Lys]; –	11/?	26.5	10.8 ± 8.5	Classic PKU
15	p. [Ile65Thr]; –	33/?	30	12.1 ± 9.9	Classic PKU
16	p. [Arg261Gln]; –	44/?	11.6	6.5 ± 4.3	Moderate/mild PKU
17	p. [Leu348Val]; –	25/?	20	8.4 ± 6.6	Classic PKU
18	p. [Ala403Val]; [Val388Met]	47	5.2	5.3 ± 2.3	Non‐PKU HPA
19	p. [Arg261Gln]; [p. Asp415Asn]	58	6.9	5.3 ± 1.4	Non‐PKU HPA
20	p. [Ile65Thr]; –	33/?	5.7	–	Non‐PKU HPA
21	p. [Val388Met]; –	28/?	6.3	6.0 ± 1.8	Non‐PKU HPA
22	p. [Arg261Gln]; –	44/?	7.0	7.1 ± 2.1	Non‐PKU HPA

*Note*: PRA: predicted residual activity for PAH (%) according to in vitro expression studies (COS and E. coli expression system).

^a^ Mean and standard deviation of Phe levels during the treatment or follow‐up period. Variants D84Y, L242F and IVS10+1 g>a do not present in vitro expression studies. Despite blood Phe values below 10 mg/dL at diagnosis, patients 4, 6, 8 and 9 in this table were classified as phenylketonurics, as they presented Phe levels compatible with PKU during treatment and biochemical monitoring (plasma Phe level >10 mg/dL).

## 4 DISCUSSION

The aim of the present study was to characterize pathogenic variants in the *PAH* gene and establish a correlation between genotype‐biochemical phenotype in patients with PKU from the state of Pará, Brazil. Table 3 shows the comparison of allelic frequencies of the most prevalent variants in the present study, with other Brazilian studies (p‐value >0.05) (Acosta et al., 2001; Santos et al., 2008; Silva et al., 2003). The p. Ile65Thr, IVS10‐11G>A and p. Val388Met variants showed real differences compared to other Brazilian studies.

**TABLE 3:** Comparison of allele frequencies of the most prevalent variants in the present study with other Brazilian studies.

Author	Year	Place	Sample (*n*)	p. Ile65Thr variant frequency (%)	*p*‐value
This study	2022	North (PA)/Brazil	32	4.7	
Acosta et al.	2001	Southeast (SP)/Brazil	292	3.5	0.8982
Silva et al.	2003	South (RS and SC)/Brazil	30	18.2	0.0033*
Santos et al.	2008	Southeast (MG)/Brazil	78	5.7	0.4011

Author	Year	Place	Sample (*n*)	p. Arg261Gln variant frequency (%)	*p*‐value
This study	2022	North (PA)/Brazil	32	7.9	
Acosta et al.	2001	Southeast (SP)/Brazil	292	12.2	0.2707
Silva et al.	2003	South (RS and SC)/Brazil	30	7.3	0.7980
Santos et al.	2008	Southeast (MG)/Brazil	78	16.0	0.0789

Author	Year	Place	Sample (*n*)	IVS10‐11G>A variant frequency (%)	*p*‐value
This study	2022	North (PA)/Brazil	32	7.9	
Acosta et al.	2001	Southeast (SP)/Brazil	292	17.4	0.0275*
Silva et al.	2003	South (RS and SC)/Brazil	30	1.8	0.1018
Santos et al.	2008	Southeast (MG)/Brazil	78	15.3	0.1006

Author	Year	Place	Sample (*n*)	p. Val388Met variant frequency (%)	*p*‐value
This study	2022	North (PA)/Brazil	32	6.3	
Acosta et al.	2001	Southeast (SP)/Brazil	292	9.1	0.2126
Silva et al.	2003	South (RS and SC)/Brazil	30	7.3	0.6226
Santos et al.	2008	Southeast (MG)/Brazil	78	21.1	0.0008*

* Indicates statistically significant.

Hillert et al. (2020) analyzed databases and identified a total of 758 different pathogenic variants in the *PAH* gene. The three most prevalent variants were p. Arg408Trp (22.2%), IVS10‐11G>A (6.4%) and p. Arg261Gln (5.5%) (Hillert et al., 2020). According to *PAH*vdb, the p. Arg408Trp has been the most reported variant in several molecular studies already performed. This pathogenic variant has a wide distribution in Europe, with high frequency (50%) in the northern and eastern European regions (Pérez et al., 1996; Zschocke, 2003). In the present study, this variant was found at a low frequency (1.5%). The p. Arg261Gln and IVS10‐11G>A variants were the most frequent pathogenic variants in the present study. The IVS10‐11G>A is the second most prevalent variant recorded in *PAH*vdb (Ferreira et al., 2021). The IVS10‐11G>A variant is distributed throughout Europe, with frequencies ranging from 1 to 25%, with the highest frequencies observed in Mediterranean populations (Ferreira et al., 2021; Hillert et al., 2020; Zschocke, 2003). The variants identified in this study, p. Val388Met, p. Arg261Gln and IVS10‐11G>A, are among the most prevalent variants found in the region of the European Iberian Peninsula (Neto, Laranjeira, et al., 2018).

In addition to the most frequent variants, the p. Asp84Tyr (1.5%), p. Arg158Gln (1.5%), p. Leu249Phe (1.5%), p. Leu348Val (1.5%), p. Tyr414Cys (1.5%) and p. Asp415Asn (1.5%) variants were also identified in other Brazilian studies: p. Asp84Tyr (frequency in Minas Gerais—1.2% and Rio de Janeiro—1.5%); p. Arg158Gln (frequency in São Paulo—3.5%; Minas Gerais—0.64%; South Region of Brazil—2.4% and Rio de Janeiro—2.5%); p. Leu249Phe (frequency in Minas Gerais—1.9% and Rio de Janeiro—2.0%); p. Leu348Val (frequency in Minas Gerais—3.2%; São Paulo—1.3% and Rio de Janeiro—2.9%); p. Tyr414Cys (frequency in São Paulo—1.3% and Rio de Janeiro—2.5%); p. Asp415Asn (frequency in Rio de Janeiro—1.0%). The other variants (p. Pro69Ser, p. Leu242Phe, p. Glu280Lys, p. Thr278Ile, IVS10+1G>A and p. Ala403Val) were identified only in patients with HPA in the state of Pará. Some of the most reported pathogenic variants in previous Brazilian studies and in *PAH*vdb, such as the IVS12n+1G>A, p. Arg261Ter and p. Arg252Trp were not identified in the present study (Desviat et al., 1995, 1999; Rivera et al., 1998; Zschocke, 2003).

Variants in the *PAH* gene vary widely among populations, mainly attributed to the population characteristics of each region (Guldberg et al., 1998; Pérez et al., 1999; Yang & Drummond‐Borg, 2001; Zschocke, 2003). In Brazil, studies carried out in the South Region, in the states of São Paulo, Minas Gerais and Rio de Janeiro, showed different mutational distributions, although in all of them, variants commonly found in the European region of the Iberian Peninsula prevail. Acosta et al. (2001) and Santos et al. (2008) demonstrated the predominance of the European contribution to the origin of the various pathogenic variants that cause PKU found in the Brazilian population, especially those originating in the Iberian Peninsula (currently formed by Portugal, Spain, Andorra and Gibraltar). In the present study, a heterogeneous mutational spectrum was also observed, in which the most frequent variants (p. Ile65Thr, IVS10‐11G>A, p. Arg261Gln and p. Val388Met) are probably of Iberian origin, and a study involving an analysis of haplotypes in the *PAH* gene is necessary to confirm this hypothesis.

The wide variety of genotypes and compound heterozygosity is a reflection of molecular heterogeneity at the *PAH* locus. In most of the studies performed, 15 or more different pathogenic variants associated with hyperphenylalaninemia due to PAH deficiency were observed, and their distribution and frequency were different between populations. Despite this molecular heterogeneity, several studies have revealed the occurrence of few frequent variants and a large number of rare variants in most populations analyzed (Desviat et al., 1995; Dianzani et al., 1995; Rivera et al., 1998; Scriver, 2007; Zschocke, 2003).

This heterogeneity was observed in the present study, through the identification of 17 different pathogenic variants associated with HPA caused by PAH deficiency. No new molecular changes were detected in this study. In other studies, carried out in Brazil, a large number of different variants were also observed: Acosta et al. (2001) in São Paulo identified 38 different variants; in the Southern region of Brazil, 22 distinct variants were found (Silva et al., 2003); in Minas Gerais, 30 different variants were identified (Santos et al., 2008) and in Rio de Janeiro, the spectrum included 37 pathogenic variants (Neto, Laranjeira, et al., 2018).

In this study, 30% (Five pathogenic variants and one nonpathogenic variant) of the variants were identified in the region comprising exon 7 and its adjacent regions. A high frequency of variants in this region has been observed in several other studies (Dianzani et al., 1995; Hillert et al., 2020; Okano et al., 1998; Rivera et al., 1998). It was also observed that 80% of the identified variants affect the enzymatic catalytic domain; that is, they are located between exons 5 and 12 of the *PAH* gene, which is in agreement with literature data (Erlandsen et al., 2003; Erlandsen & Stevens, 1999; Hillert et al., 2020).

Nine patients had pretreatment Phe levels above 20 mg/dL, characterized as classic PKU. Variations in PKU (pretreatment Phe levels between 10 and 20 mg/dL), which include moderate and mild forms of the disease, were observed in 12 patients in the sample. In most studies, severe (null) variants in homozygous or compound heterozygous patients (the presence of two different severe variants) are associated with classic PKU, while mild variants give rise to enzymes with considerable residual activity.

Patients who have a mild variant and a severe variant usually manifest mild or moderate PKU phenotypes (Guldberg et al., 1998). In this study, patients with the classic PKU phenotype had variants that confer low or zero enzyme activity (PRA between 0% and 11%), and the highest pretreatment Phe levels were observed in these patients. Variants associated with a moderate or mild in vitro enzyme phenotype (PRA between 25% and 72%) were identified in patients with lower pretreatment Phe levels (moderate/mild PKU and non‐PKU HPA).

In the present study, variants p. Ile65Thr and p. Arg261Gln were identified in patients with biochemical features of classic PKU, mild PKU and non‐PKU HPA. Studies report that the p. Ile65Thr variant may be associated with any type of HPA due to PAH deficiency (Erlandsen et al., 2003; Guldberg et al., 1998). As this variant is located in a regulatory region of the enzyme, the phenotypes may present a wide spectrum. The p. Arg261Gln variant is located in the catalytic domain of the enzyme and may be found in phenotypes associated with any type of HPA due to PAH deficiency (Erlandsen et al., 2003; Guldberg et al., 1998). The p. Val388Met variant was identified in patients with mild PKU and non‐PKU HPA. In addition to the p. Asp415Asn and p. Ala403Val variants, in non‐PKU HPA patients, three variants were identified (p. Ile65Thr, p. Arg261Gln and p. Val388Met) that are associated with PKU patients.

Most patients with PAH deficiency are compound heterozygotes, making it difficult to establish the severity of each variant and its contribution to the expression of the phenotype. Due to the phenotypic heterogeneity found in patients with HPA due to PAH deficiency, the clinical use of genotype analysis is generally limited (Wettstein et al., 2015).

Of the 32 patients with HPA due to PAH deficiency analyzed in this study, 9 patients had their genotype defined (two mutant alleles identified) and of these 7 are compound heterozygotes and two homozygotes for the IVS10‐11G>A variant. In the present study, it was not possible to predict the phenotype from the genotype in 3 patients (c. [IVS10+1G>A]; p. [Pro69Ser], p. [Asp84Tyr]; [Thr278Ile] and p. [Leu242Phe]; [p. Arg158Gln]) since these genotypes have not yet been associated with any phenotypic class. This lack of association is related to the presence of rare and poorly studied variants (absence of in vitro variant expression studies) and shows that knowledge of the genotype may have a limited role in some cases.

A large number of studies in different populations have shown correlations and inconsistencies between genotype and metabolic phenotype (Acosta et al., 2001; Guldberg et al., 1998; Himmelreich et al., 2018; Okano et al., 1991; Scriver, 2007; Silva et al., 2003; Wettstein et al., 2015). Okano et al. (1991) analyzed the genotype of 104 phenylketonuric patients, based on the residual enzymatic activity resulting from 8 different experiments and correlated with the biochemical phenotype of these patients. This analysis showed a significant modification between the genotype and the metabolic phenotype, although the enzymatic activity of the patients was shown from in vitro expression studies, thus demonstrating an in vivo situation in a simplified way (Okano et al., 1991).

In Europe, Guldberg et al. (1998) identified and analyzed the genotypes of 686 patients from 7 European treatment centres. Based on the analysis of these patients' variants, it was possible to predict the metabolic phenotype of 650 patients. In 79% of the patients the expected phenotype was different from the observed phenotype. Guldberg et al. (1998) suggested that differences in methods used for variants detection and/or for classifying phenotypes may explain a large number of inconsistencies in the genotype–phenotype relationship.

Table 4 shows the comparison of the genotype–phenotype correlation observed in this study with literature data (*PAH*vdb) in six patients with known genotypes. The analysis of literature data showed that in some patients, the same genotype may be associated with several different metabolic phenotypes. In the present study, the genotype c. [IVS10‐11G>A]; [IVS10‐11G>A] was associated with pretreatment Phe levels between 10 and 20 mg/dL (moderate/mild PKU) and with pretreatment Phe levels above 20 mg/dL (classic PKU) (patients 2 and 4 from Table 4).

**TABLE 4:** Genotype–phenotype correlation in PKU patients from the state of Pará: observed phenotype (present study) and expected phenotype (*PAH*vdb).

Patients	Genotype	Observed phenotype	Expected phenotype
1	c. [IVS10‐11G>A]; p. [Tyr414Cys]	Classic PKU	Moderate/mild PKU
2	c. [IVS10‐11G>A]; [IVS10‐11G>A]	Moderate/mild PKU	Classic PKU
3	p. [Ile65Thr]; [Val388Met]	Moderate/mild PKU	Moderate/mild PKU
4	c. [IVS10‐11G>A]; [IVS10‐11G>A]	Classic PKU	Classic PKU
5	p. [Ala403Val]; [Val388Met]	Non‐PKU HPA	Non‐PKU HPA
6	p. [Arg261Gln]; [p. Asp415Asn]	Non‐PKU HPA	Non‐PKU HPA

Abbreviation: *PAH*vdb, phenylalanine hydroxylase gene locus‐specific database.

According to *PAH*vdb, the genotype c. [IVS10‐11G>A]; [IVS10‐11G>A] (null homozygote: a variant responsible for undetectable *PAH* activity) confers an expected classic PKU phenotype, but patient 2 in Table 4 had a Phe level equal to 15 mg/dL at the time of diagnosis (pretreatment), characterizing a metabolic phenotype of moderate/mild PKU. This fact reinforces the presence of inconsistencies in the genotype–phenotype relationship in the present study, also observed in patient 1 of Table 4 (genotype: c. [IVS10‐11G>A]; p. [p. Tyr414Cys]), who presented a different metabolic phenotype than expected on the basis of literature data.

In summary, the molecular analysis carried out in PKU patients in the state of Pará in the North Region of Brazil revealed a heterogeneous mutation pattern, in which the most frequent molecular changes are variants in the *PAH* gene commonly found in studies carried out with patients from the European Iberian Peninsula. In other regions of Brazil, these variants are also among the most frequent *PAH* variants identified. The continuation of this study could contribute to a more complete analysis of the *PAH* gene with the objective of detecting the variants in those patients with not completely defined genotypes (1 mutant allele identified) and in new patients diagnosed in the North Region of Brazil.

## AUTHOR CONTRIBUTIONS

PEBF: performed the experiments, analyzed data and wrote the manuscript. RSA: acquisition of clinical and biochemical data. AKRS: performed the molecular tests. LCSS: assisted with funding, conception, design of the study and final approval of the version to be submitted.

## FUNDING INFORMATION

This research was financially supported by the Laboratory of Inborn Errors of Metabolism of the Federal University of Pará in the performance of laboratory tests.

## CONFLICT OF INTEREST STATEMENT

The authors report no declarations of interest.

## ETHICS STATEMENT

This study was performed in accordance with the Declaration of Helsinki and was approved by the Research Ethics Committee of the João de Barros Barreto University Hospital (protocol number 1111).

## References

[mgg32224-bib-0001] Acosta, A. X. , Silva, W. A. , Carvalho, T. M. , Gomes, M. , & Zago, M. A. (2001). Mutations of the phenylalanine hydroxylase (PAH) gene in Brazilian patients with phenylketonuria. Human Mutation, 17(2), 122–130. 10.1002/1098-1004(200102)17:2<122::AID-HUMU4>3.0.CO;2-C 11180595

[mgg32224-bib-0002] Blau, N. , Hennermann, J. B. , Langenbeck, U. , & Konecki, U. L. (2011). Diagnosis, classification, and genetics of phenylketonuria and tetrahydrobiopterin (BH4) deficiencies. Molecular Genetics and Metabolism, 104(Suppl), S2–S9. 10.1016/j.ymgme.2011.08.017 21937252

[mgg32224-bib-0003] Blau, N. , Shen, N. , & Carducci, C. (2014). Molecular genetics and diagnosis of phenylketonuria: State of the art. Expert Review of Molecular Diagnostics, 14(6), 655–671. 10.1586/14737159.2014.923760 24882081

[mgg32224-bib-0004] Blau, N. , Spronsen, V. F. , & Levy, L. H. (2010). Phenylketonuria. Lancet, 376, 1417–1427. 10.1016/S0140-6736(10)60961-0 20971365

[mgg32224-bib-0005] Desviat, L. R. , Pérez, B. , De Lucca, M. , Cornejo, V. , Schmidt, B. , & Ugarte, M. (1995). Evidence in Latin America of recurrence of V388M, a phenylketonuria mutation with high in vitro residual activity. American Journal of Human Genetics, 57(2), 337–342. 7668259 PMC1801570

[mgg32224-bib-0006] Desviat, L. R. , Pérez, B. , Gámez, A. , Sánchez, A. , García, M. J. , Martínez‐Pardo, M. , Marchante, G. , Bóveda, D. , Baldellou, A. , Arena, J. , Sanjurjo, P. , Fernández, A. , Cabello, M. L. , & Ugarte, M. (1999). Genetic and phenotypic aspects of phenylalanine hydroxylase deficiency in Spain: Molecular survey by regions. European Journal of Human Genetics, 7(3), 386–392. 10.1038/sj.ejhg.5200312 10234516

[mgg32224-bib-0007] Dianzani, I. , Giannattasio, S. , Sanctis, L. , Alliaudi, C. , Lattanzio, P. , Vici, C. D. , Burlina, A. , Burroni, M. , Sebastio, G. , Carnevale, F. , Guzzeta, V. , Marra, E. , Camaschella, C. , & Ponzone, A. (1995). Characterization of phenylketonuria alleles in the Italian population. European Journal of Human Genetics, 3(5), 294–302. 10.1159/000472313 8556304

[mgg32224-bib-0008] Erlandsen, H. , Patch, M. G. , Gamez, A. , Straub, M. , & Stevens, R. C. (2003). Structural studies on phenylalanine hydroxylase and implications toward understanding and treating phenylketonuria. Pediatrics, 112(6 Pt 2), 1557–1565. 14654665

[mgg32224-bib-0009] Erlandsen, H. , & Stevens, R. C. (1999). The structural basis of phenylketonuria. Molecular Genetics and Metabolism, 68(2), 103–125. 10.1006/mgme.1999.2922 10527663

[mgg32224-bib-0010] Ferreira, F. , Azevedo, L. , Neiva, R. , Sousa, C. , Fonseca, H. , Marcão, A. , Rocha, H. , Carmona, C. , Ramos, S. , Bandeira, A. , Martins, E. , Campos, T. , Rodrigues, E. , Garcia, P. , Diogo, L. , Ferreira, A. C. , Sequeira, S. , Silva, F. , Rodrigues, L. , … Vilarinho, L. (2021). Phenylketonuria in Portugal: Genotype‐phenotype correlations using molecular, biochemical, and haplotypic analyses. Molecular Genetics & Genomic Medicine, 9(3), e1559. 10.1002/mgg3.1559 33465300 PMC8104178

[mgg32224-bib-0011] Guldberg, P. , Rey, F. , Zschocke, J. , Romano, V. , François, B. , Michiels, L. , Ullrich, K. , Hoffmann, G. F. , Burgard, P. , Schmidt, H. , Meli, C. , Riva, E. , Dianzani, I. , Ponzone, A. , Rey, J. , & Guttler, F. (1998). A European multicenter study of phenylalanine hydroxylase deficiency: Classification of 105 mutations and a general system for genotype‐based prediction of metabolic phenotype. American Journal of Human Genetics, 63(1), 71–79. 10.1086/301920 9634518 PMC1377241

[mgg32224-bib-0012] Gundorova, P. , Stepanova, A. A. , Kuznetsova, A. I. , Kutsev, I. S. , & Polyakov, V. A. (2019). Genotypes of 2579 patients with phenylketonuria reveal a high rate of BH4 non‐responders in Russia. PLoS One, 14(1), e0211048. 10.1371/journal.pone.0211048 30668579 PMC6342299

[mgg32224-bib-0013] Hillert, A. , Anikster, Y. , Belanger‐Quintana, A. , Burlina, A. , Burton, B. K. , Carducci, C. , Chiesa, A. E. , Christodoulou, J. , Đorđević, M. , Desviat, L. R. , Eliyahu, A. , Evers, R. A. F. , Fajkusova, L. , Feillet, F. , Bonfim‐Freitas, P. E. , Giżewska, M. , Gundorova, P. , Karall, D. , Kneller, K. , … Blau, N. (2020). The genetic landscape and epidemiology of phenylketonuria. American Journal of Human Genetics, 107(2), 234–250. 10.1016/j.ajhg.2020.06.006 32668217 PMC7413859

[mgg32224-bib-0014] Himmelreich, N. , Shen, N. , Okun, J. G. , Thiel, C. , Hoffmann, G. F. , & Blau, N. (2018). Relationship between genotype, phenylalanine hydroxylase expression and in vitro activity and metabolic phenotype in phenylketonuria. Molecular Genetics and Metabolism, 125(1–2), 86–95. 10.1016/j.ymgme.2018.06.011 30037505

[mgg32224-bib-0015] Lidksy, A. S. , Robson, K. J. , Thirumalachary, C. , Barker, P. E. , Ruddle, F. H. , & Woo, S. L. (1984). The PKU locus in man is on chromosome 12. American Journal of Human Genetics, 36(3), 527–533. 6547271 PMC1684467

[mgg32224-bib-0016] Neto, V. E. , Filho, M. S. H. , Monteiro, B. C. , Carvalho, M. L. , Tonon, T. , Vanz, P. A. , Schwartz, D. V. I. , & Ribeiro, G. M. (2018). Quality of life and adherence to treatment in early‐treated Brazilian phenylketonuria pediatric patients. Brazilian Journal of Medical and Biological Research, 51(2), e6709. 10.1590/1414-431X20176709 PMC5731329 29267500

[mgg32224-bib-0017] Neto, V. E. , Laranjeira, F. , Quelhas, D. , Ribeiro, I. , Seabra, A. , Mineiro, N. , Carvalho, L. M. , Lacerda, L. , & Ribeiro, G. M. (2019). Genotype‐phenotype correlations and BH_4_ estimated responsiveness in patients with phenylketonuria from Rio de Janeiro, Southeast Brazil. Molecular Genetics & Genomic Medicine, 7(5), e610. 10.1002/mgg3.610 30829006 PMC6503030

[mgg32224-bib-0018] Neto, V. E. , Laranjeira, F. , Quelhas, D. , Ribeiro, I. , Seabra, A. , Mineiro, N. , Carvalho, M. L. , Lacerda, L. , & Ribeiro, G. M. (2018). Mutation analysis of the *PAH* gene in phenylketonuria patients from Rio de Janeiro, Southeast Brazil. Molecular Genetics & Genomic Medicine, 6(4), 575–591. 10.1002/mgg3.408 29749107 PMC6081236

[mgg32224-bib-0019] Okano, Y. , Eisensmith, R. C. , Guttler, F. , Lichter‐Konecki, U. , Konecki, D. S. , Trefz, F. K. , Dasovich, M. , Wang, T. , Henriksen, K. , Lou, H. , & Woo, S. L. (1991). Molecular basis of phenotypic heterogeneity in phenylketonuria. The New England Journal of Medicine, 324(18), 1232–1238. 10.1056/NEJM199105023241802 2014036

[mgg32224-bib-0020] Okano, Y. , Minoru, A. , Youngbo, K. , Yasuaki, N. , Yutaka, H. , Toshiaki, O. , & Isshiki, G. (1998). Molecular characterization of phenylketonuria in Japanese patients. Human Genetics, 103(5), 613–618. 10.1007/s004390050877 9860305

[mgg32224-bib-0021] Pérez, B. , Desviat, L. R. , De Lucca, M. , Cornejo, V. , Raimann, E. , & Ugarte, M. (1999). Molecular characterization of phenylalanine hydroxylase deficiency in Chile. Mutations in Brief n^0^ 243. Online. Human Mutation, 13(6), 503. 10.1002/(SICI)1098-1004(1999)13:6<503::AID-HUMU12>3.0.CO;2-I 10408782

[mgg32224-bib-0022] Pérez, B. , Desviat, L. R. , De Lucca, M. , Schimidt, B. , Loghin‐Grosso, N. , Giugliani, R. , Pires, R. F. , & Ugarte, M. (1996). Mutation analysis of phenylketonuria in South Brazil. Human Mutation, 8(3), 262–264. 10.1002/(SICI)1098-1004(1996)8:3<262::AID-HUMU10>3.0.CO;2-0 8889586

[mgg32224-bib-0023] Rivera, I. , Leandro, P. , Lichter‐Konecki, U. , Almeida, I. T. , & Lechner, M. C. (1998). Population genetics of hyperphenylalaninaemia resulting from phenylalanine hydroxylase deficiency in Portugal. Journal of Medical Genetics, 35(4), 301–304. 10.1136/jmg.35.4.301 9598724 PMC1051278

[mgg32224-bib-0024] Santos, L. L. , Magalhães‐Castro, M. , Fonseca, C. G. , Starling, A. L. P. , Januário, J. N. , Aguiar, M. J. B. , & Carvalho, M. R. S. (2008). PKU in Minas Gerais state, Brazil: Mutation analysis. Annals of Human Genetics, 72(Pt 6), 774–779. 10.1111/j.1469-1809.2008.00476.x 18798839

[mgg32224-bib-0025] Scriver, C. R. (2007). The PAH gene, phenylketonuria, and a paradigm shift. Human Mutation, 28(9), 831–845. 10.1002/humu.20526 17443661

[mgg32224-bib-0026] Scriver, C. R. , & Kaufman, S. (2001). Hyperphenylalaninemia: Phenylalanine hydroxylase deficiency. In C. R. Scriver , A. L. Beaudet , W. S. Sly , & D. Valle (Eds.), The metabolic and molecular bases of inherited disease (8th ed., pp. 1667–1724). McGraw‐Hill.

[mgg32224-bib-0027] Silva, L. C. S. , Carvalho, T. S. , Silva, F. B. , Morani, L. , Fachel, A. A. , Pires, R. F. , Refosco, L. F. , Desnick, R. , Giuglian, R. , & Pereira, M. L. S. (2003). Molecular characterization of phenylketonuria in South Brazil. Molecular Genetics and Metabolism, 79, 17–24. 10.1016/s1096-7192(03)00032-5 12765842

[mgg32224-bib-0028] Wettstein, S. , Underhaug, J. , Perez, B. , Marsden, D. B. , Yue, W. W. , Martinez, A. , & Blau, N. (2015). Linking genotypes database with locus‐specific database and genotype‐phenotype correlation in phenylketonuria. European Journal of Human Genetics, 23(3), 302–309. 10.1038/ejhg.2014.114 24939588 PMC4326710

[mgg32224-bib-0029] Willians, A. R. , Mamotte, D. S. C. , & Burnett, R. J. (2008). Phenylketonuria: An inborn error of phenylalanine. Clinical Biochemist Reviews, 29(1), 31–41. 18566668 PMC2423317

[mgg32224-bib-0030] Yang, Y. , & Drummond‐Borg, M. (2001). Molecular analysis of phenylketonuria (PKU) in newborns from Texas. Human Mutation, 17(6), 523. 10.1002/humu.1141 11385716

[mgg32224-bib-0031] Zschocke, J. (2003). Phenylketonuria mutations in Europe. Human Mutation, 21(4), 345–356. 10.1002/humu.10192 12655544

[mgg32224-bib-0032] Zschocke, J. (2010). Molecular basis of phenylketonuria: From genotype to clinical management. Annales Nestlé (English ed.), 68, 48–52. 10.1159/000312811

